# Aggregation of GPS, WLAN, and BLE Localization Measurements for Mobile Devices in Simulated Environments

**DOI:** 10.3390/s19071694

**Published:** 2019-04-09

**Authors:** Kamil Książek, Krzysztof Grochla

**Affiliations:** Institute of Theoretical and Applied Informatics, Polish Academy of Sciences, ul. Baltycka 5, 44-100 Gliwice, Poland; kksiazek@iitis.pl

**Keywords:** BLE, GPS, WiFi, localization, positioning system, location aggregation, IPS

## Abstract

There are multiple available technologies to find the location of a mobile device, such as the Global Positioning System (GPS), Bluetooth Low-Energy beacons (BLE), and Wireless LAN (WLAN) localization. We propose a novel method to estimate the location of a moving device by aggregating information from multiple positioning systems into a single, more precise location estimation. The aggregated location is calculated as the place in which the product of the probability density functions (PDF) of individual methods has the maximum value. The experimental probability density functions of the three analyzed technologies are fitted by gamma distributions based on error histograms found in the literature and measurement data. The location measurements of the individual technologies are provided at different time instants, so the weighted product of the PDFs is used to improve aggregation accuracy. The discrete event-simulation model was used to evaluate the aggregation method with the Gauss–Markov mobility model. Simulations demonstrated that the calculated aggregated location was more accurate than any of the methods taken as the input, and average error was decreased by almost 13% compared to an arithmetic mean of the three considered localization methods, and by more than 36% compared to the single method with the highest accuracy.

## 1. Introduction

Localization is an important function of mobile devices. Smartphones, tablets, and wireless network devices provide not only communication functions but, thanks to being able to measure one’s current location, can also help users navigate and provide location-based services [1]. Mobile devices are now equipped with multiple radio interfaces that can be used to estimate device location. Bluetooth and GPS receivers exist in almost every smartphone. There are also many indoor positioning systems (IPSs) based on available WiFi. Most positioning systems are based on a single technology, but there are also hybrid positioning systems available for finding the location of a mobile device using several different positioning technologies. According to the Cisco Visual Networking Index [2], in 2018 there were approximately 20 billion networking devices, of which 24% were smartphones. This shows that there is an enormous market of devices capable of using different indoor and outdoor positioning techniques.

Most positioning systems are based on measurements coming from one technology. GPS technology has become the most widely deployed solution [3], with billions of devices using it for outdoor-location measurements. There were trials for also using GPS for indoor positioning, see, e.g., Reference [4], but due to the high attenuation of walls, in most applications GPS was not suitable for IPS systems. Multiple other technologies for location estimation were developed based on infrared, magnetism, radio waves, sound, or visual light information. Between those, two technologies became very popular: WiFi indoor positioning [5], due to the high availability of WiFi devices, and Bluetooth Low Energy (BLE), thanks to battery-powered beacons. Nevertheless, the GPS remains a main source of location functions, with other, less accurate methods mainly being used when GPS signal is unavailable.

Most smartphones are now equipped with GPS, WiFi, and Bluetooth, allowing the use any of those three technologies for positioning. The selection method that location techniques use in a specific location is still an open question. The simplest solution (but widely used) is to use the most accurate available technology, for example, using the GPS when signal is available, and location-based 3G and WiFi AP received signal strength indication (RSSI) measurements when the GPS signal is unavailable. Operating systems for mobile devices, such as Google Android or iOS, provide location API functions to retrieve the current location [6,7]. Nevertheless, when positioning systems based on WiFi or BLE are deployed, it is an unresolved challenge to merge the information provided by various sources. While WLAN and BLE positioning are mainly used indoors, there are many cases in which they can also be used outdoors. For example, Zou et al. [8] described an energy-efficient scheme for indoor- and outdoor-location-based services running on the mobile devices based on iBeacon technology; Zhang et al. [9] used crowdsourcing to develop WiFi fingerprint positioning. WiFi signals are omnipresent in cities and BLE beacons are deployed to support mobile applications with guide functions in places like castles [10] or cities [11].

There are a few examples of hybrid location-estimation techniques that gather information from two radio technologies to estimate location. Baniukevic et al. [12] employed both Wi-Fi and Bluetooth in one system and used fingerprinting, which requires gathering information about signal maps for both technologies. A similar approach was to create a fusion of WiFi and Bluetooth fingerprinting in which RSS measurements were compared against the fingerprints of the points within the previously determined zone, using Bluetooth to increase precision [13]. Another paper [14] presented a smartphone indoor-positioning engine named HIPE that merged information from different smartphone sensors to resolve position estimates with the application of Hidden Markov Model (HMM) problems, a grid-based filter, and the Viterbi algorithm. There are many other solutions that use a special-purpose hardware unit to observe the WLAN-RSSI with the merger of other radio-signal measurements for indoor positioning by fingerprinting [15]. However, all these techniques depend on the precision of the signal level maps, which are costly to obtain and are required to provide measurements for each location technology.

The aggregation of location information provided by various sources is not a simple task, as location measurements are provided in different time instants and intervals (e.g., most GPS interfaces by default provide location data once per second, while BLE beacons may be transmitted every 300 ms, and WLAN beacons are by default transmitted every 100 ms). Aggregation needs to take into account measuring-device movements between moments at which the location measurements are gathered. A device moving even at quite a low speed may move between two consecutive measurements by a few meters (e.g., a bicycle moving with a speed of 18 km/h traverses 5 m between two GPS location measurements). In Reference [16], the authors proposed an energy-efficient prediction-based method for target tracking, taking into account the distance from the predicted location and remaining node energy. Nevertheless, the proposed method is based only on a single source of location information.

The fusion of the outcome of two location techniques was previously discussed [17]. Bayesian filtering and data-level fusion with the merger of a simulated annealing algorithm was used to obtain a position estimate. In another paper [18], GPS information was merged with an inertial navigation system using Kalman filters for effective land-vehicle localization. Another solution is to use one technology to improve the accuracy of another, for example, in Reference [19], Ultra wideband (UWB) nodes used GPS signal to establish a fine degree of synchronization, resulting in more accurate positional determination via UWB. It allows to provide very precise measurements, but requires specialized hardware and data processing. All of the above listed techniques are based on the specifics of two (or more) particular location technologies and do not allow to merge the outcome of different location techniques in a generic way, taking into account the difference in the measurement time of the technologies. Another issue is that error characteristics for GPS, WLAN, and BLE are significantly different, which makes it almost impossible to use the average of many measurements into a single, more accurate location estimation.

The rest of the paper is organized as follows: in Section 2 we describe the proposed method of location-measurement aggregation, in Section 3 we describe the methodology of error-estimation aggregation, and in Section 4 we show the simulation results. We finish with our conclusions in Section 5.

## 2. Proposed Location-Aggregation Method

The main idea of the proposed location-aggregation algorithm is to discover a point in which the probability of finding a mobile node is the largest at a given moment of time. For this purpose, it is necessary to estimate the distribution errors of each available system used for localization and to find a location in which probability multiplication of the presence according to all location-information sources (e.g., GPS, WLAN, or BLE) is highest. In addition, when the device is moving, more recent measurements more precisely represent its current position. This is due to the error caused by the change of location, that is, the movement of the device between the time at which the measurement was made and the current time is smaller. This is taken into account by weights, which are described in detail in Section 2.5.

### 2.1. Error-Distribution Fitting

Simulation of the behavior of analyzed methods requires fitting probability density functions to real data as, to the best of the authors’ knowledge, there are no papers showing the analytical probability distribution functions of WLAN or BLE localization systems. The main purpose is the estimation of the error distributions of individual location-estimation methods. During the fitting procedure, a well-known heuristic algorithm called particle-swarm optimization [20] was applied [21].

This method imitates a swarm intelligence (e.g., fish, birds). During consecutive iterations, points move to new places in the domain considering their own best position in history, the best position of the swarm, and randomness, according to the following equations:

$${\vec{x_{i}}}^{\left(j+1\right)}={\vec{x_{i}}}^{\left(j\right)}+{\vec{v_{i}}}^{\left(j+1\right)},$$

(1) $${\vec{v_{i}}}^{\left(j+1\right)}=c_{1}\cdot {\vec{v_{i}}}^{\left(j\right)}+c_{2}\cdot r\left(0,1\right)\left({\vec{x_{i}}}^{\left(best\right)}-{\vec{x_{i}}}^{\left(actual\right)}\right)+c_{3}\cdot r\left(0,1\right)\left({\vec{x_{i}}}^{\left(globalBest\right)}-{\vec{x_{i}}}^{\left(actual\right)}\right),$$

where ${\vec{x_{i}}}^{\left(j\right)}$, ${\vec{v_{i}}}^{\left(j+1\right)}$ are the localization and velocity of the *i*-th point during the *j*-th iteration, respectively. ${\vec{x_{i}}}^{\left(best\right)}$ is the best position (with the highest value of an evaluation function) of *i*-th point, ${\vec{x_{i}}}^{\left(globalBest\right)}$ is the best position of the swarm and ${\vec{x_{i}}}^{\left(actual\right)}$ is the current position of *i*-th point. $c_{i}$, i∈{1,2,3} are coefficients, r(0,1) is a pseudorandom number from interval [0,1). In this case, $c_{1}=0.729$, $c_{2}=c_{3}=1.49445$, according to Reference [22].

On the basis of real data, three gamma distributions were estimated, associated with the analyzed methods. The hypothesis that gamma distribution is a good approximation of distribution errors was taken because the histograms of the location methods present in the literature show that error probability distributions are left-skewed and have long right tails. The calculations presented in following subsections confirm these arguments.

### 2.2. GPS

In the case of the GPS, the probability density function was generated by using data coming from the Federal Aviation Administration in Washington [23]. A sample was composed of more than 255 million elements. The data provide measurements with horizontal errors in meters, divided into 0.1 m width intervals in the range from 0 to 13.6 m:

(2) $$S=\{\left(0.0,n_{0}\right),\left(0.1,n_{1}\right),\left(0.2,n_{2}\right),\ldots ,\left(13.6,n_{137}\right)\}.$$

where $n_{i}$ is the number of elements in *i*-th bin of the histogram.

Such data are normalized and used to fit the probability density function of errors. Normalization means the division of each grid point by the total number of occurrences and multiplication by interval length (0.1 m) to ensure that $\int_{0}^{\infty }f_{GPS}\left(x;k,\theta \right)dx=1$. It is required to go from a discrete to a continuous distribution. Thus, 

(3) $$\forall j\in \left\{0,1,2,\ldots ,m\;\right\}\;\;\;n_{j}:=0.1\cdot \frac{n_{j}}{\sum_{k=0}^{m}n_{k}}.$$

**Figure 1:**
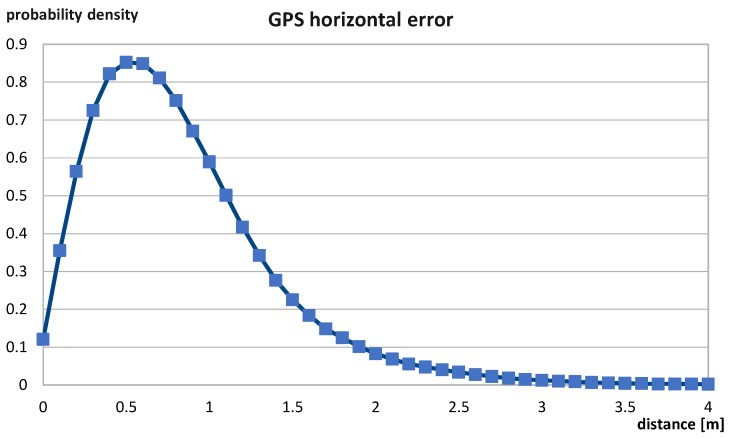
Global Positioning System (GPS) horizontal error distribution based on real data.

In the presented case, m=137. Figure 1 shows an empirical probability density function of GPS horizontal error based on the described data. The analytical function needs to be matched to these data to represent the GPS error in the model. To find such a function, the optimization procedure was used to find the function parameters that provided the smallest root-mean-squared error. The goal function of the optimization has following form:

(4) $$RMSE=\sqrt{\sum_{i=1}^{m}{\left(f_{GPS}\left(x_{i};k,\theta \right)-y_{i}\right)}^{2}},$$

where $f_{GPS}\left(x_{i};k,\theta \right)$ is a value of the gamma distribution with a shape parameter *k* and a scale parameter θ in *i*-th point of grid, $y_{i}$ is the probability of a specific error in *i*-th point of grid, i∈{1,2,…,m}.

The appropriate probability density function was estimated by using particle-swarm optimization (30 particles, 1000 epochs). The exact results are available in Table 1. It turned out that gamma distribution with a shape parameter k=2.331727 and a scale parameter θ=0.370786 was a proper choice for fitting empirical values from GPS. This distribution was also proposed in Referehce [24]. It yields to the following equation:

(5) $$f_{GPS}\left(x\right)=\left\{\begin{array}{cc} 8.4984\;e^{-2.69697x}x^{1.33173} & x>0\\ 0 & x\leq 0 \end{array}\right.$$

**Figure 2:**
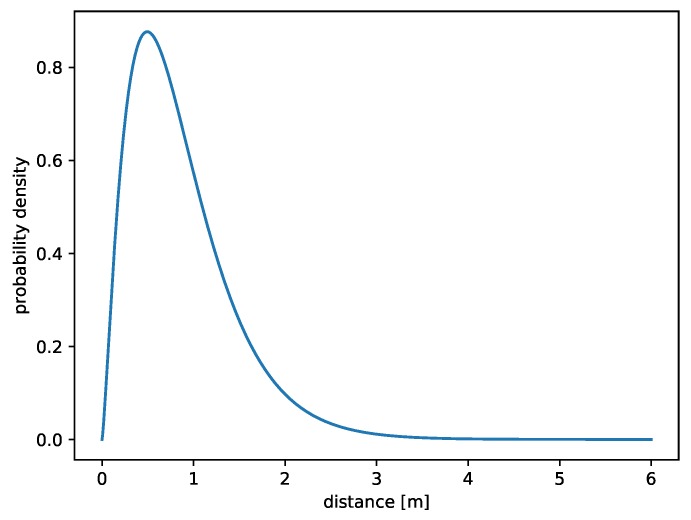
Gamma distribution (2.33,0.37) fitted to GPS horizontal errors.

A graph of the probability density function of gamma distribution with adjusted parameters can be seen in Figure 2. GPS is the most accurate from three described methods, but it gives results only every 1000 ms.

### 2.3. WLAN

The second applied method for collecting localization data is based on Wireless LAN fingerprinting [25]. It was proposed for indoor localization and has lower accuracy than the GPS, but is also available in many outdoor locations, where multiple WiFi APs are deployed. It is also possible to get a frequent output of measurements because WiFi beacons are transmitted every 100 ms. Similarly, particle-swarm optimization was used to designate the gamma-distribution parameters: gamma distribution represents well error characteristics in positioning methods based on fingerprinting [26]. According to values of cumulative distribution function (CDF) representing the localization error of a method based on kernel principal-component analysis (Figure 9 in Reference [25]), following evaluation function $\Phi_{WLAN}$ that was created:

(6) $$\begin{array}{c} \Phi_{WLAN}=|\Gamma_{k,\theta }\left(0.5\right)-0.17|+|\Gamma_{k,\theta }\left(1\right)-0.42|+|\Gamma_{k,\theta }\left(1.5\right)-0.6|+|\Gamma_{k,\theta }\left(2\right)-0.7|\\ +|\Gamma_{k,\theta }\left(2.5\right)-0.84|+|\Gamma_{k,\theta }\left(3\right)-0.94|+|\Gamma_{k,\theta }\left(3.5\right)-1.0|. \end{array}$$

Heuristic methods indicated that k=1.755533 (a shape parameter) and θ=0.848053 (a scale parameter) were good choices for gamma distribution. The obtained results enable to create a pattern for probability density function:

(7) $$f_{WLAN}\left(x\right)=\left\{\begin{array}{cc} 1.45115\;e^{-1.17917x}x^{0.755533} & x>0\\ 0 & x\leq 0 \end{array}\right.$$

**Figure 3:**
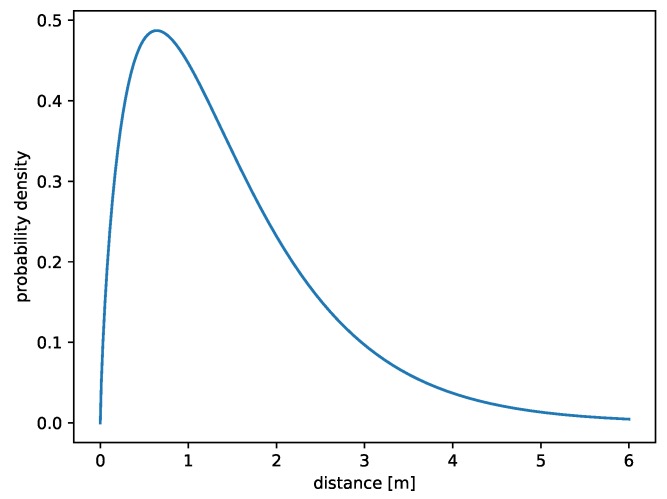
Gamma distribution (1.27852,0.74295) representing errors of a method based on Wireless LAN.

Equation (7) graph is shown in Figure 3. It is possible to see that the most likely error value in the case of the method based on WLAN is about one meter.

### 2.4. BLE Beacons

The third method used for localization of a mobile node concerns BLE beacons. Error distribution was estimated on the basis of error values shown in Table 6.6 in Reference [27]. Assessment was not as good as in previous cases because the available data were not so detailed, but it was enough for reliable simulations. Again, gamma-distribution parameters were fitted using particle-swarm optimization. Evaluation function $\Phi_{BLE}$, designated, for minimization is as follows:

(8) $$\Phi_{BLE}=|\Gamma_{k,\theta }\left(2\right)-0.231|+|\Gamma_{k,\theta }\left(3\right)-0.692|+|\Gamma_{k,\theta }\left(4\right)-0.846|+|\Gamma_{k,\theta }\left(5\right)-0.846|,$$

where *k* and θ are the shape and scale parameters of gamma distribution, respectively. The least value of Equation (8) was achieved by using k=6.48936 and θ=0.44277, which led to the following probability density function:

(9) $$f_{BLE}\left(x\right)=\left\{\begin{array}{cc} 0.700026\;e^{-2.25849x}x^{5.48936} & x>0\\ 0 & x\leq 0 \end{array}\right.$$

**Figure 4:**
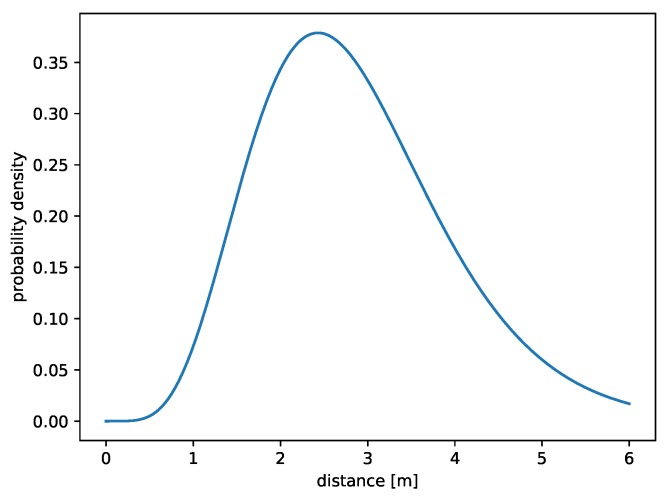
Gamma distribution (6.48936,0.44277) representing errors of a method based on Bluetooth Low-Energy (BLE) beacons.

A graph of Equation (9) is presented in Figure 4. This method is the least accurate of the three described approaches, thus providing the biggest localization error of, in most cases, more than two meters. Despite this, thanks to the low cost of the beacons, localization based on BLE is becoming increasingly popular.

**Figure 5:**
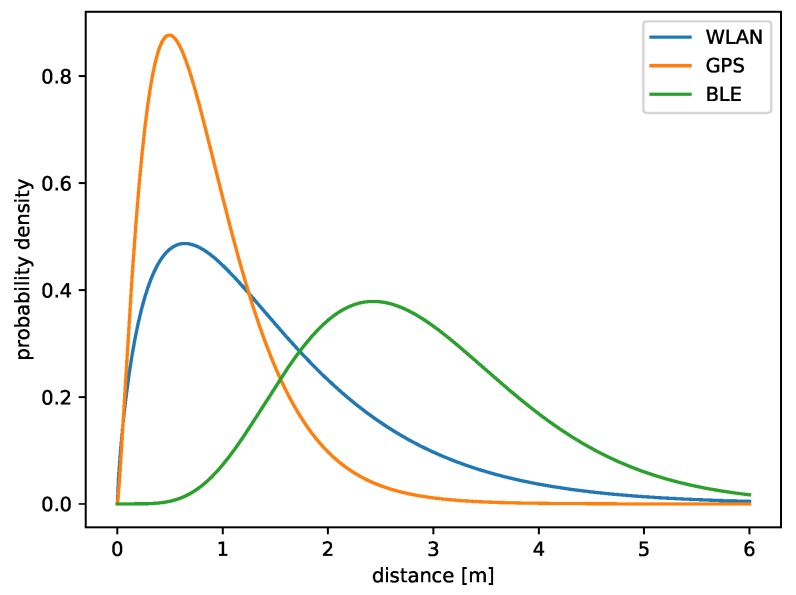
Gamma distributions corresponding to localization methods used in simulations.

The distribution errors of three presented methods were estimated. They are shown together in Figure 5. Thanks to such an estimation technique, a bigger impact on a value of Φ(x,y) has more precise modules: the maximum of $f_{GPS},f_{WLAN}$, and $f_{BLE}$ is equal to 0.88, 0.49, and 0.38, respectively.

### 2.5. Location Aggregation

The location-aggregation algorithm uses the multiplication rule for independent events to find a location, for which the coincidence of errors for each method is highest. This approach is similar to the maximum-likelihood method. There are multiple sources of location information, in the analyzed case, coming from GPS, WLAN, and BLE positioning systems. The point at which the product of each of the methods’ error PDF is highest was selected as a candidate. It was assumed that this is the location in which the measured device is most likely present. The goal of the algorithm is realized through maximizing a value of evaluation function Φ(x,y):

(10) $$\begin{array}{c} \Phi \left(x,y\right)=\sum_{i=1}^{n_{1}}\left(1-(t-t_{i})\right)f_{GPS}\left(d_{GPS}^{i}\left(x,y\right)\right)+\sum_{i=1}^{n_{2}}\left(1-(t-t_{i})\right)f_{WLAN}\left(d_{WLAN}^{i}\left(x,y\right)\right)\\ +\sum_{i=1}^{n_{3}}\left(1-(t-t_{i})\right)f_{BLE}\left(d_{BLE}^{i}\left(x,y\right)\right), \end{array}$$

where $d_{METHOD}^{i}\left(x,y\right)$ is the Euclidean distance between *i*-th point of a given method (GPS, WLAN, or BLE) and a calculated point (x,y). Thus,

(11) $$\underset{(x,y)\in \Omega }{arg max}\Phi \left(x,y\right)=\left\{\left(x_{best},y_{best}\right)\in \Omega :\forall \left(x,y\right)\in \Omega \;\;\;\Phi \left(x,y\right)\leq \Phi \left(x_{best},y_{best}\right)\right\}$$

is a solution of the issue. Because of the estimation of errors by using gamma distributions, Φ(x,y) is unimodal function, so arg maxΦ(x,y) has a unique solution that is the method output. It is now necessary to precisely describe the components of Φ(x,y).

Location measurement by different methods is done at different time instants. In this paper, three sources of location information of the mobile node are considered: GPS, WLAN, and BLE beacons. It is assumed that GPS sends location data every 1000 ms, WLAN has an interval of 100 ms, and BLE beacons transmit data every 300 ms. Those are the default intervals at which following devices output location measurements. Furthermore, it is the smallest interval in which location measurements are possible. The aggregated location is calculated every 250 ms.

Within one time window (250 ms), it is possible to obtain two or three measurements from the system based on WLAN, zero or one from GPS, and, similarly, zero or one measurement of location information from BLE beacons. Hence, all the collected data from previous computations were used in the calculations. If any system (GPS or BLE), however, did not provide information in the previous window, the latest measurement from such a method was attached to the calculations. So, in the presented case, $n_{1}=1$, $n_{2}=2$ or 3 and $n_{3}=1$. Furthermore, the meaning of coefficients $1-(t-t_{i})$ needed to be clarified (*t* is a current time and $t_{i}$ is the time at which *i*-th measurement was delivered). These are weighting factors that define the importance of subsequent summands. The smaller a difference $\left(t-t_{i}\right)$ is, the higher the value that the corresponding coefficient has. This means that the newest data have the biggest impact on the final result.

To find the estimated location, the maximum of Φ(x,y) function needs to be found. For this purpose, two approaches were compared: the grid method, and the local-search heuristic algorithm. The first is based on grid generation: the domain of the search is divided into squares with an equal size (for the sake of the analysis, 1 cm ×1 cm was used). Then, the value of Φ(x,y) is calculated in each square. Coordinates in which the square with the maximum value of Φ(x,y) is located are an output of this method. While this approach is computationally complex, it is simple in implementation, and allows to achieve the desired accuracy by changing the size of the square.

The second technique, local search, is a relatively simple and widely used optimization method [28]. It has lower computational complexity, as it does not require calculating the values of Φ(x,y) in so many points. One should define an initial point (in this paper, it is an arithmetic mean of *m* points obtained from the following methods, where *m* is the number of summands in Φ according to the described approach). During consecutive iterations, new points are randomly generated in the neighborhood of the best one according to the following equation:

(12) $$x_{i}^{j}=x_{best}^{j}+\alpha^{j}\cdot U\left(-1,1\right),\;\;\;j=1,2,$$

where $x_{i}^{j}$ is the *j*-th coordinate of the *i*-th point, $\vec{x_{best}}=\left(x_{best},y_{best}\right)$ is a point with the highest value of evaluation function until the last iteration, $\vec{\alpha }=\left(\alpha_{1},\alpha_{2}\right)$ is a vector of parameters, and U(−1,1) is a pseudorandom number from uniform distribution in interval [−1,1). Then, if $\Phi \left(x,y\right)\geq \Phi \left(x_{best},y_{best}\right)$, the point is considered as the best so far. At the end of each iteration, the values of $\vec{\alpha }$ are reduced (exploitation of domain).

Algorithm 1 presents a pseudocode of the proposed location-aggregation method.

**Algorithm 1** Pseudocode of the localization-aggregation method**Input:** Points indicated by used methods with timestamps: GPS $\left\{\left(x_{GPS}^{1},y_{GPS}^{1}\right),\ldots ,\left(x_{GPS}^{n_{1}},y_{GPS}^{n_{1}}\right)\right\}$; $\left\{t_{GPS}^{1},\ldots ,t_{GPS}^{n_{1}}\right\}$, WLAN $\left\{\left(x_{WLAN}^{1},y_{WLAN}^{1}\right),\ldots ,\left(x_{WLAN}^{n_{2}},y_{WLAN}^{n_{2}}\right)\right\}$, $\left\{t_{WLAN}^{1},\ldots ,t_{WLAN}^{n_{2}}\right\}$, BLE $\left\{\left(x_{BLE}^{1},y_{BLE}^{1}\right),\ldots ,\left(x_{BLE}^{n_{3}},y_{BLE}^{n_{3}}\right)\right\}$, $\left\{t_{BLE}^{1},\ldots ,t_{BLE}^{n_{3}}\right\}$, distributions of errors of GPS ($f_{GPS}$), WLAN ($f_{WLAN}$) and ($f_{BLE}$), present timestamp *t* **Output:** Coordinates of the optimum with value of function Φ(x,y) (Equation (10)) Calculate range of calculations:$x_{min}=min\left\{x_{GPS}^{1},\ldots ,x_{GPS}^{n_{1}},x_{WLAN}^{1},\ldots ,x_{WLAN}^{n_{2}},x_{BLE}^{1},\ldots ,x_{BLE}^{n_{3}}\right\}$,$x_{max}=max\left\{x_{GPS}^{1},\ldots ,x_{GPS}^{n_{1}},x_{WLAN}^{1},\ldots ,x_{WLAN}^{n_{2}},x_{BLE}^{1},\ldots ,x_{BLE}^{n_{3}}\right\}$,$y_{min}=min\left\{y_{GPS}^{1},\ldots ,y_{GPS}^{n_{1}},y_{WLAN}^{1},\ldots ,y_{WLAN}^{n_{2}},y_{BLE}^{1},\ldots ,y_{BLE}^{n_{3}}\right\}$,$y_{max}=max\left\{y_{GPS}^{1},\ldots ,y_{GPS}^{n_{1}},y_{WLAN}^{1},\ldots ,y_{WLAN}^{n_{2}},y_{BLE}^{1},\ldots ,y_{BLE}^{n_{3}}\right\}$.Find a point (x,y) where $x\in [x_{min},x_{max}]$, $y\in [y_{min},y_{max}]$, with the maximum value of evaluation function Φ(x,y) (10) according to the grid method, local search, or another optimization method.

## 3. Aggregation-Accuracy Analysis

### 3.1. Movement Model

**Figure 6:**
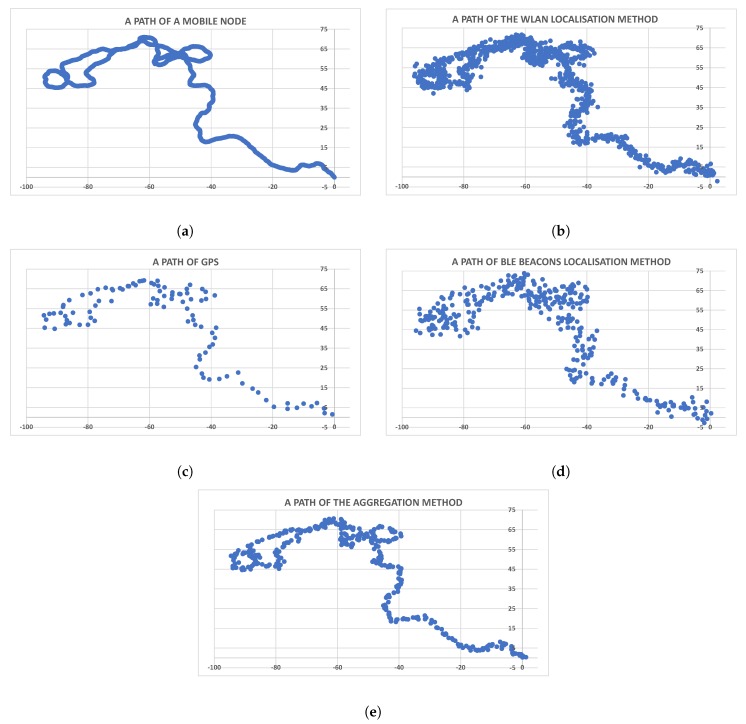
Exemplary path determined by the specific methods. (**a**) A real path of the mobile node (according to the Gauss–Markov mobility model). (**b**) A path appointed by a method based on WLAN. (**c**) Path appointed by GPS. (**d**) Path appointed by a method based on BLE beacons. (**e**) Path appointed by the localization-aggregation method.

Proper simulation of an object’s movement (for instance, a vehicle in a hall) requires an appropriate mobility model. The Gauss–Markov mobility model [29] was selected as a model that represents changes in both movement speed and direction, preserving some inertia [30]. It is represented by the following equations:

(13) $$\begin{array}{c} {\vec{v}}^{t}=\vec{v}+\left({\vec{v}}^{t-1}-\vec{v}\right)\cdot \beta +\left(1-\beta \right)\cdot \vec{v}\cdot N\left(0,1\right),\\ {\vec{\varphi }}^{t}={\vec{\varphi }}^{t-1}+\left(1-\beta \right)\cdot \vec{\varphi }\cdot N\left(0,1\right), \end{array}$$

where ${\vec{v}}^{t}$ is a speed at time *t*, $\vec{v}$ is mean speed, β is a parameter (β∈[0,1]), and N(0,1) is a pseudorandom number from normal distribution with a mean equal to 0 and standard deviation equal to 1. β is a parameter connected with the memory of a node. If β=0, a new speed is completely different than the previous one. Setting β=1 causes the node to move according to the uniform rectilinear motion. Intermediate values (β∈(0,1)) influence movement variability (for instance, β close to 1 implies a large impact of a previous step). Proper choice of parameters like $\beta ,\vec{v}$ and $\vec{\varphi }$ facilitates modeling object behavior. An exemplary path of an object moving according to this mobility model is shown in Figure 6a.

### 3.2. Simulation Model

For the evaluation of the described method, a simulation model was developed. The simulations were implemented in OMNeT++ 5.4.1 with the assumption of the following conditions:

- a mobile node moves according to the Gauss–Markov mobility model with parameters: β=0.9, $\bar{v}=3\frac{m}{s}$, $\bar{\varphi }=\frac{\pi }{2}$;
- position of the mobile node changes every 5 ms;
- there exist three localization systems: GPS, WLAN, and BLE beacons; as mentioned before, and they give information about localization every 1000 ms, 100 ms, and 300 ms, respectively;
- for successive methods, errors to the actual position are added according to the corresponding distribution error (for instance, GPS errors are pseudorandom values from gamma distribution with shape parameter 2.33 and scale parameter 0.37);
- a position of the mobile node is calculated every 250 ms.

**Figure 7:**
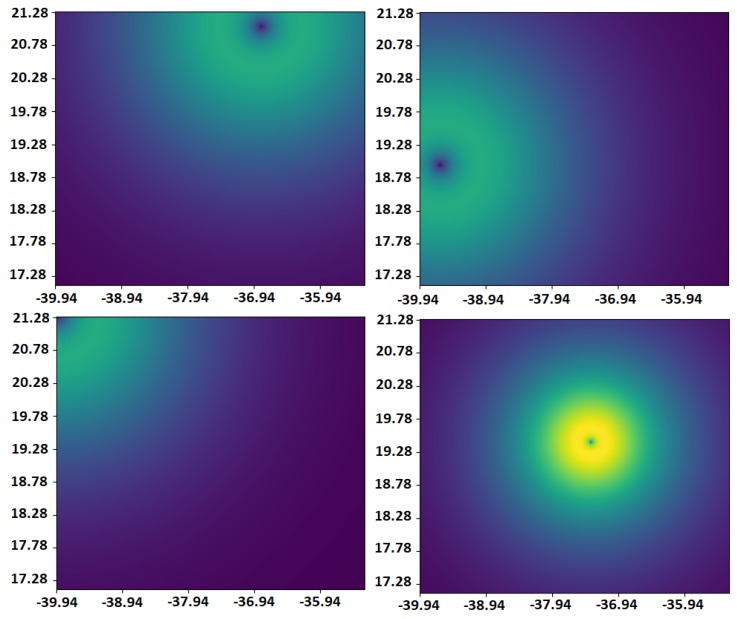

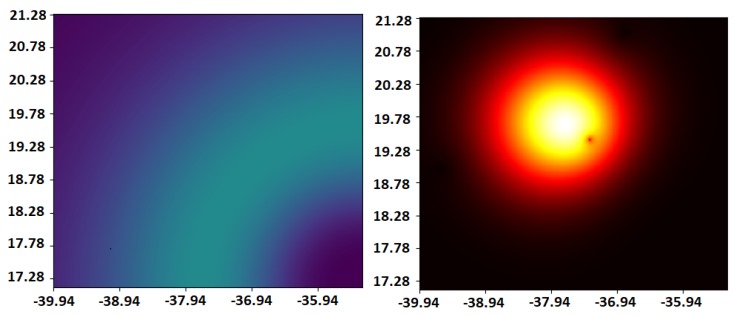
Visualization of data-aggregation method. On the first row are two measurements from the WLAN localization method. On the second row are localization data from the WLAN module (third measurement) and GPS. On the third row are data from a method based on BLE beacons and the localization-aggregation algorithm. Node is located at (−37.99,19.53).

**Figure 8:**
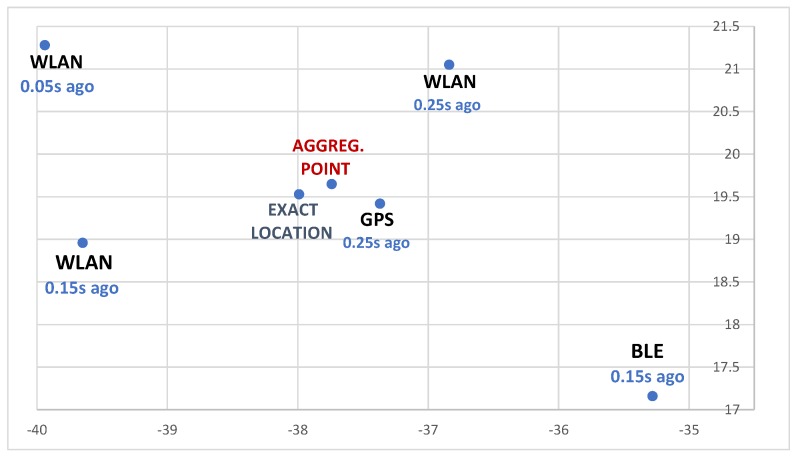
Exemplary point plot and their timestamps given by all methods according to scenario from Figure 7.

Tests lasted for 48 simulation hours, which generated a sample with almost 700,000 cases of position calculations. Figure 7 presents a visualization of an exemplary case, with measurements of particular methods as in Figure 8. The lighter a color is, the greater the probability of finding a node. Distributions are consistent with the previous section. The most accurate method (GPS) had the largest impact on the final result, but was, of course, balanced by weight coefficients. The last picture demonstrates an aggregate score. Presented images illustrate an idea of the described approach well—it is like the maximum likelihood method.

## 4. Results and Discussion

### 4.1. Aggregation of WLAN, GPS, and BLE Localization

**Figure 9:**
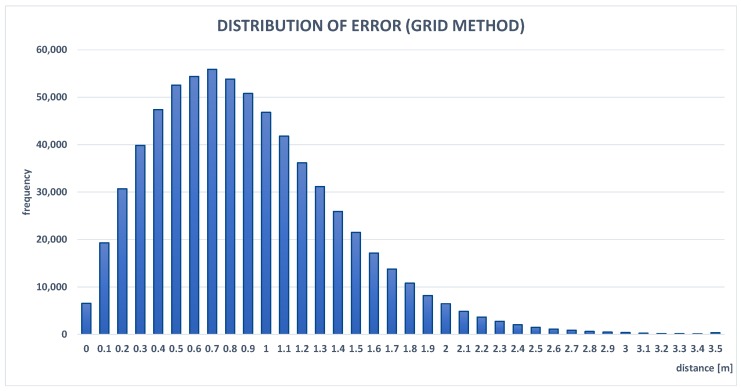
Experimental probability distribution function of error for location aggregation, with maximum selected using grid method.

**Figure 10:**
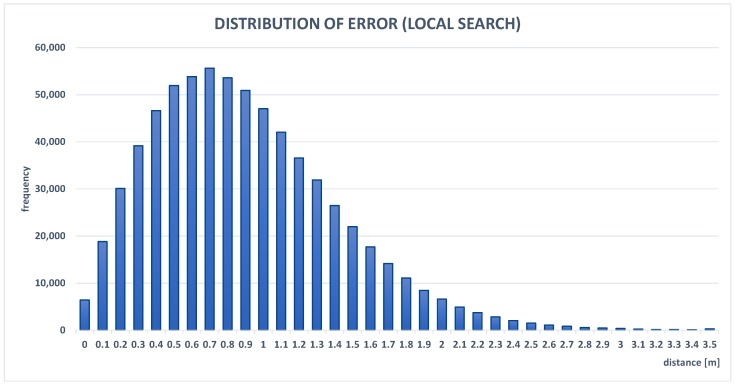
Experimental probability distribution function of error for location aggregation, with maximum selected using local search.

Results presented in Table 2 confirmed that the presented localization-aggregation method achieved the best accuracy. Average errors for the grid method and local search were less than one meter. Local search was significantly faster than the grid method because it does not require calculating function values in the whole domain. Despite this, such an approach is very efficient and has similar results to the grid method. The simple alternative to the proposed algorithm, i.e., the arithmetic mean that collects localization data from the last time window (250 ms) is more than 14% worse than the aggregated location. Each individual localization method (WLAN, GPS, and BLE beacons) achieved significantly worse scores. GPS was most accurate from the presented modules, but it rarely provides information, only every 1000 ms, which introduces a large error related to the location change of the mobile device. Although WLAN causes larger errors than GPS, it can more often send localization data. The worst accuracy of the three compared systems was by BLE beacons, with an average error slightly less than three meters. The results show that the simple selection of the location provided by the positioning system with highest accuracy causes an error higher by more than 50% than the aggregated location. A comparison between the real path of the mobile node, positions approximated by the aggregation algorithm, and other localization methods is shown in Figure 6. This confirms that the presented technique is the most effective, as its path was the closest to the real one. The GPS path is the most accurate, but this method has large intervals between consecutive points. Figure 9 and Figure 10 present the error distributions for the two optimization methods used. Distributions show a very low probability of errors higher than two meters, which proves the stability of the aggregation method. Furthermore, their similarity proves that local search can be an efficient alternative for the grid method.

### 4.2. Aggregation of Only WLAN and GPS

In addition, measurements without the least effective method (based on BLE beacons) were performed. The received results, shown on Table 3, indicate that even the weakest system has a positive impact on the final point (average error was slightly worse without BLE beacons). Understandably, the accuracy of the arithmetic mean was about 0.06 m better than before (this statistic is sensitive to deviations).

### 4.3. Adaptation to Different Positioning System Error Characteristics

The accuracy characteristics of localization information provided by the GPS, WLAN, and BLE positioning systems may vary. In particular, the error characteristics provided by positioning systems based on fingerprinting may change depending on the physical conditions in which it is used, such as the number of beacons and the distances between beacons. The aggregation method proposed in this paper is universal, as data can be collected from any *n* modules with different error characteristics. The method can be adapted to incorporate error distribution, measured in real life by repeating the procedure of gathering the error histogram measured for a particular localization method and fitting error distribution using particle-swarm optimization, as described in Section 2.2 for GPS. Similarly, the proposed aggregation method can easily be adapted to different localization methods (e.g., UWB) by extending the evaluation function through addition of the estimation of distribution error of the other considered method.

## 5. Conclusions and Future Work

The aggregation of localization data is an important issue, as more positioning systems are being deployed and may be used to improve the accuracy of location tracking. In this paper, a localization-aggregation method that allows the calculation of a mobile-node position was proposed. The usefulness of this approach was proven through discrete event simulation. It is possible to improve the accuracy of localization estimation and decrease the error by one-third through merging information from individual methods between moments in which they transmit localization data. A first step relies on assessing errors generated by subsequent modules. Then, it is necessary to create the evaluation function according to the described steps and find a place where the maximum of this function is located. The evaluation was performed for GPS, WLAN, and BLE beacon location systems. The simulation results confirmed that the aggregation algorithm in the paper could increase the localization accuracy of a mobile node by 13% compared to an arithmetic mean. The proposed method is more general and can be adapted to incorporate other indoor- or outdoor-positioning systems through the adaptation of the error distribution of the different localization methods, and through the application of weight coefficients corresponding to reception time. The fitting verification to the error distributions and the accuracy of the method in real-world experiments, as well as the integration of the aggregation with filtering, such as Kalman filters, which can further improve accuracy, are future research challenges. 

## Figures and Tables

**Table 1:** Fitting a distribution by using particle-swarm optimization—experiment results.

Distribution	Root-Mean-Squared Error (*RMSE*) Values	Parameters
Gamma	0.01636	shape=2.331727,scale=0.370786
Weibull	0.01710	scale=0.910326,scale=1.68668
Normal	0.03513	mean=0.654757,std.deviation=0.479675
Cauchy	0.05591	location=0.618981,scale=0.362026
Exponential	0.11877	rate=0.749887

**Table 2:** Accuracy of aggregation of all location methods: GPS, WLAN, and BLE.

Description	Average Error (m)	Relative Error	Standard Deviation
Grid method	0.94242	100.00%	0.51959
Local Search	0.94883	100.68%	0.51900
Arithmetic mean	1.07947	114.54%	0.59607
GPS	1.49522	158.66%	0.87496
WLAN	1.50353	159.54%	1.12203
BLE beacons	2.90712	308.47%	1.18136

**Table 3:** Accuracy of GPS and WLAN aggregation (without BLE beacon data).

Description	Average Error (m)	Relative Error	Standard Deviation
Grid method	0.95197	100.00%	0.53865
Local Search	0.96055	100.90%	0.52843
Arithmetic mean	1.01307	106.42%	0.62801
GPS	1.49814	157.37%	0.87623
WLAN	1.50363	157.95%	1.12050
